# Refining the Quality Indicator Set from the Dutch Audit for Treatment of Obesity to Ensure Usefulness in Clinical Practice

**DOI:** 10.1007/s11695-025-07898-2

**Published:** 2025-06-12

**Authors:** Floris F.E. Bruinsma, Simon W. Nienhuijs, Ronald S.L. Liem, Jan Willem M. Greve, Perla J. Marang-van de Mheen

**Affiliations:** 1 Maastricht University Medical Centre, Maastricht, Netherlands; 2 Scientific Bureau, Dutch Institute for Clinical Auditing, Leiden, Netherlands; 3 Catharina Hospital, Eindhoven, Netherlands; 4 Groene Hart Hospital, Gouda, Netherlands; 5 Nederlandse Obesitas Kliniek, The Hague, Netherlands; 6 Weight Doctors Nederland, Quole, Waalre, Netherlands; 7 Delft University of Technology, Delft, Netherlands

**Keywords:** Quality indicators, Metabolic bariatric surgery, Quality indicator set, Systematic evaluation, Quality measures

## Abstract

**Background:**

Quality of metabolic bariatric surgery (MBS) care is often monitored by national registries using quality indicators (QIs), but data collection takes up considerable time and costs. QIs are mostly introduced merely based on expert opinion. Therefore, the study’s aim was to systematically evaluate whether all QIs from the Dutch Audit for Treatment of Obesity (DATO) are still relevant and useful to initiate quality improvement initiatives.

**Methods:**

Twenty-four QIs were evaluated using hospital data from 2022 to 2023. To test whether QIs measured the same quality of care aspect (parsimony and relevance), correlations of QI pairs were examined using Pearson correlation coefficients. Usefulness to identify improvement opportunities was considered limited when variance is ≤ 0.001 without any outliers identified, indicating that the QI could be retired. Actionability was assessed through line graphs of hospital performance over the years.

**Results:**

Eleven QIs were highly correlated to other QIs, particularly the follow-up and weight loss indicators at 2 and 4 years, and therefore lacked added value. The weight loss QIs showed minimal variance and were adjusted by increasing the threshold to achieving ≥ 25% total weight loss. Multiple QIs showed improving trends and thereby their actionability, most pronounced for postoperative complications. The final QI set measured three constructs with good validity: Cronbach’s alpha values 0.53 (safety), 0.70 (treatment effectiveness), and 0.43 (follow-up process).

**Conclusion:**

Through a systematic evaluation of the DATO QI set, a smaller set of 13 QIs was shown to capture the same relevant information to improve MBS care.

**Supplementary Information:**

The online version contains supplementary material available at 10.1007/s11695-025-07898-2.

## Introduction

Over the past decade, there has been a growing focus on comparing healthcare quality among caregivers to uncover opportunities for quality improvement [1]. Quality registries have been introduced in many countries with the aim of providing feedback to healthcare providers on how their performance compares with others, which can be used as a starting point for improvement initiatives [2–5]. Part of this feedback is delivered through quality indicators (QIs), which are measures to assess hospital performance. Besides their use for feedback purposes, these indicators are also employed to promote transparency, providing information to stakeholders such as patients and healthcare insurers [6], and are typically listed on governmental websites [7, 8].

For metabolic bariatric surgery (MBS), over 30 quality registries already exist worldwide [9], often with their own QI set for evaluating the standard of delivered care [4], Individual QIs are usually proposed by an expert panel [10, 11] and measure specific aspects of the quality of care delivered. Although it is not uncommon for individual QIs to be assessed on criteria such as reliability and validity [12–14], the QI set as a whole is rarely evaluated [15, 16]. Indicators can be both process measures, such as follow-up rates, and outcome measures, such as weight loss and complication rates. The Dutch MBS registry – the Dutch Audit for Treatment of Obesity (DATO) – has defined several QIs that are publicly reported [17], and hospitals are evaluated based on their results by national authorities and insurers, who may ask for clarification when hospitals are underperforming. However, certain QIs may no longer be useful, for instance, because they do not reveal differences between hospitals anymore or measure nearly identical aspects of care as other indicators, suggesting that the number of indicators—and consequently the registration burden—could be reduced.

This study aims to systematically evaluate the QI set from DATO on parsimony and relevance, usability, and actionability, following a previously suggested method [18]. The results can help to refine the QI set, ensuring it minimizes the registration burden while maintaining its ability to identify areas for improvement.

## Methods

### Data Collection

All data were obtained from DATO [17], a mandatory national MBS quality registry that has been collecting hospital and patient data since 2015. Approximately 12,000 patients are added to the registry annually [8]. All twenty bariatric institutes in the Netherlands contribute to this registry, and on-site data verification has shown high data validity [19]. The study was approved by the DATO scientific committee. As DATO operates as an opt-out registry under Dutch regulations, informed consent was not required.

### Quality Indicators

The DATO QI set is based on 8 parameters, which are subdivided into 27 QIs that are transparent to the public [7]. Two of these are volume indicators, ensuring that hospitals perform sufficient MBS procedures to guarantee adequate care, in line with Dutch protocols and guidelines [20]. These indicators were deemed conditional to provide good quality of care but not to measure the quality of care delivered and therefore not further evaluated. One indicator measures the percentage response regarding quality of life (QoL) questionnaires before and after surgery, but not the questionnaire results itself, as a different questionnaire was recently introduced and awaits more research on how the results could reflect quality. Therefore, 24 QIs were included in the evaluation and considered the QI set throughout the article (see Table 1). All QIs are measured among patients undergoing primary MBS, unless specified differently. One QI involves a composite endpoint referred to as “textbook outcome,” indicating a patient is discharged from the hospital within 2 days and has not experienced any postoperative complications or readmissions within the first 30 days. Due to the significant impact of the COVID-19 pandemic on the accessibility of MBS [21], which may have affected QI scores during those years, only hospital performance scores for 2022 and 2023 were included to minimize bias, unless stated otherwise.

**Table 1:** Description of included quality indicators from the Dutch Audit for Treatment of Obesity.

**QI**		**Description**
**1**	**Textbook outcome**	Percentage of patients receiving primary surgery meeting with the criteria for Textbook Outcome*
**2**	**Improved HbA1c**	Percentage of type 2 diabetic patients receiving primary surgery with improved HbA1c values 1 year after surgery
**3a**	**Follow-up**	Percentage of patients receiving primary surgery with completed consultation during the 1^st^ follow-up year
**3b**		Percentage of patients receiving primary surgery with completed consultation during the 2^nd^ follow-up year
**3c**		Percentage of patients receiving primary surgery with completed consultation during the 3^rd^ follow-up year
**3 d**		Percentage of patients receiving primary surgery with completed consultation during the 4^th^ follow-up year
**3e**		Percentage of patients receiving primary surgery with completed consultation during the 5^th^ follow-up year
**4a**	**Complicated course**	Percentage of patients receiving primary surgery with a severely complicated postoperative course**
**4b**		Percentage of patients receiving secondary surgery with a severely complicated postoperative course**
**5a1**	**% Total weight loss**	Percentage of patients receiving primary gastric sleeve surgery, who have achieved at least 20% TWL 1 year after surgery
**5a2**		Percentage of patients receiving primary gastric bypass surgery, who have achieved at least 20% TWL 1 year after surgery
**5a3**		Percentage of patients receiving primary surgery other than gastric sleeve or gastric bypass, who have achieved at least 20% TWL 1 year after surgery
**5b1**		Percentage of patients receiving primary gastric sleeve surgery, who have achieved at least 20% TWL 2 years after surgery
**5b2**		Percentage of patients receiving primary gastric bypass surgery, who have achieved at least 20% TWL 2 years after surgery
**5b3**		Percentage of patients receiving primary surgery other than gastric sleeve or gastric bypass, who have achieved at least 20% TWL 2 years after surgery
**5c1**		Percentage of patients receiving primary gastric sleeve surgery, who have achieved at least 20% TWL 3 years after surgery
**5c2**		Percentage of patients receiving primary gastric bypass surgery, who have achieved at least 20% TWL 3 years after surgery
**5c3**		Percentage of patients receiving primary surgery other than gastric sleeve or gastric bypass, who have achieved at least 20% TWL 3 years after surgery
**5 d1**		Percentage of patients receiving primary gastric sleeve surgery, who have achieved at least 20% TWL 4 years after surgery
**5 d2**		Percentage of patients receiving primary gastric bypass surgery, who have achieved at least 20% TWL 4 years after surgery
**5 d3**		Percentage of patients receiving primary surgery other than gastric sleeve or gastric bypass, who have achieved at least 20% TWL 4 years after surgery
**5e1**		Percentage of patients receiving primary gastric sleeve surgery, who have achieved at least 20% TWL 5 years after surgery
**5e2**		Percentage of patients receiving primary gastric bypass surgery, who have achieved at least 20% TWL 5 years after surgery
**5e3**		Percentage of patients receiving primary surgery other than gastric sleeve or gastric bypass, who have achieved at least 20% TWL 5 years after surgery

*QI* quality indicator, *HbA1c* glycated hemoglobin, *TWL* total weight loss, *gastric bypass* Roux-en-Y gastric bypass, one anastomosis gastric bypass, or ring augmented gastric bypass

*Textbook outcome is a composite endpoint, including no postoperative complications in the first 30 days, hospital discharge within 2 days, and no readmission within the first 30 days

**Complicated course = postoperative complication Clavien-Dindo grade 3+ within the first 30 days

### Evaluation Steps and Analysis

The QI set was evaluated in four steps, following a previously recommended methodology [18]. If the QI set was adjusted during one step, this revised set was included in subsequent evaluation steps.

#### Parsimony and Relevance

Highly correlated QIs are likely to measure the same aspect of quality of care, adding administrative burden without offering additional insights for quality improvement. Scatter plots were created for pairs of QIs that, based on their content, could be correlated, and Pearson correlation coefficients were determined. If a Pearson correlation coefficient was > 0.70, the QIs were inspected by the clinical audit board to explore whether they could be combined or if one indicator should be dropped from the QI set.

The following pairs of QIs were tested for possible correlation: textbook outcome and complicated postoperative course, improved HbA1c values and %TWL (as weight loss is related to improvement in diabetes [22]), improved HbA1c values and follow-up (as the venipuncture is usually combined with the outpatient visit during follow-up), follow-up at different time points, %TWL at different time points, %TWL for different procedures, and complicated postoperative course for primary and secondary surgery. This resulted in 39 pairs of QIs which were tested for correlations.

#### Usability

QIs are considered usable in clinical practice if they can identify opportunities for improvement. When there is minimal variation across hospitals on certain QIs, with all institutions performing similarly, the potential for identifying improvement opportunities based on these QIs appears limited. Following the criteria suggested by Xu et al [18], further improvement is unlikely if the variance is < 0.001, and the QI could be considered for retirement. However, if the variance is below 0.001 but significant outlier performance in one or more hospitals is observed in a funnel plot [23]—a visual graph often used in quality comparisons—the QI still demonstrates improvement potential for at least one hospital and should therefore not be abandoned yet. Therefore, the QI was considered for discontinuation if the variance remained below 0.001 for two consecutive years, and the funnel plot revealed no hospitals with outlier performance during this period.

#### Actionability

QIs that demonstrate improvement over time have shown their actionability and can be considered effectively implemented in clinical care when all hospitals approach optimal performance levels (e.g., 100%). To evaluate progress, line graphs were generated for each QI, covering the period from introduction until 2023, depicting the median QI rate, the mean QI rate for the 20% best performing hospitals, and the mean QI rate for the 10% worst performing hospitals (minimum of two hospitals). If QIs have not shown improvement since their introduction, either the QI is not actionable, or insufficient focus has been placed on improving the QI in question.

#### Construct Validity

An ideal QI set should contain information on different aspects of quality of care. While outcome information undeniably plays an important role, insights into in-hospital processes are equally valuable. Improving in-hospital processes contributes to optimizing patient care, which can, in turn, influence outcomes. When creating a QI set, careful consideration should be given to which constructs are intended to be measured, for instance, treatment safety or effectiveness. Therefore, a confirmatory factor analysis was conducted to assess the extent to which three underlying constructs were captured:

1. Safety, measured by postoperative outcomes (indicator 1 on textbook outcome, and indicators 4a and 4b on severely complicated postoperative course)
2. Effectiveness of treatment, measured by weight loss outcomes and HbA1c values for patients with diabetes (indicator 2 on improved HbA1c values 1 year after surgery, indicators 5a1–5e3 on achieving at least 20% total weight loss (TWL) at 1 to 5 years after surgery for different bariatric procedures)
3. Follow-up process (indicators 3a–3e for different points of follow-up)

Construct validity of these three constructs was evaluated by calculating Cronbach’s alpha, with alpha > 0.4 indicating adequate consistency and reliability [24].

## Results

### Parsimony and Relevance

Strong correlations (*R* > 0.7) were found for 14 of the 39 QI pairs, which involved either follow-up or total weight loss (%TWL) indicators at different time points (see Table 2, highlighted in orange). Indicators measuring the same outcome in consecutive years (e.g., the score at 1 year vs. 2 years) showed particularly strong correlations, more than those observed in non-consecutive years (e.g., 1 year vs. 3 years). Considering the follow-up indicators, year 2 correlated strongly with year 3, while year 4 correlated with both year 3 and 5. Therefore, measuring years 1, 3, and 5 alone would give a sufficiently comprehensive view of follow-up completeness, allowing the indicators for year 2 and 4 to be excluded.

Considering the %TWL indicators for different procedure types, multiple strong correlations were found between measurements in consecutive years, in particular within the QIs evaluating gastric bypass and sleeve gastrectomy. Again, if year 2 and 4 were excluded from the QI set, the remaining indicators would still provide adequate information for assessing hospital performance. The group of procedures other than sleeve gastrectomy and gastric bypass was too small to effectively compare outcomes between hospitals (all hospitals performing < 10 annually), so these QIs were proposed to be excluded. Out of the original 24 QIs, 13 were selected for further evaluation in the subsequent steps.

**Table 2.**
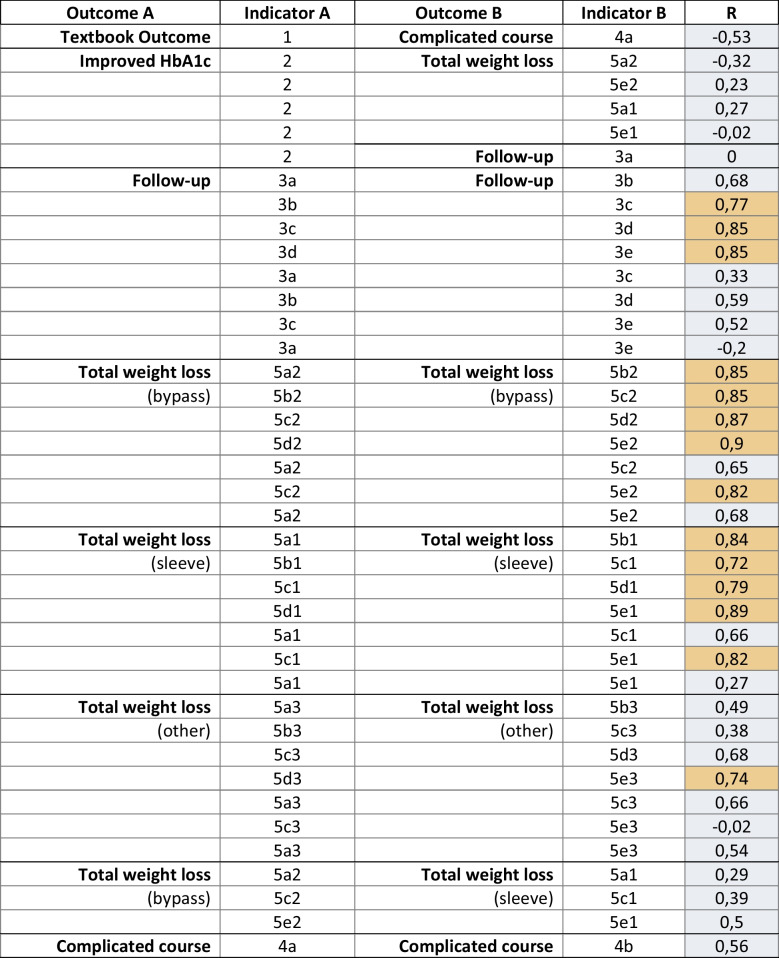
Pairs of quality indicators that were tested for correlations

*HbA1c*, glycated hemoglobin; *R*, correlation coefficient; orange, *R* > 0.7

Textbook Outcome = composite endpoint for no complications within 30 days after surgery, hospital discharge within 2 days, and no readmission within 30 days; complicated course = postoperative complication Clavien-Dindo grade 3+ within the first 30 days

### Usability

Most remaining QIs had variances greater than 0.001, indicating that they could still discriminate between hospitals and identify opportunities for improvement (Table 3). Four QIs had variances smaller than 0.001, but three of these QIs still identified hospitals with outlier performance in the funnel plot (see supplementary figures 1a-1 d). Only the QI reflecting the percentage of patients achieving at least 20%TWL 1 year after gastric bypass demonstrated both low variance and no outliers in the funnel plot, suggesting it may be redundant. In general, the QIs measuring %TWL after sleeve gastrectomy showed greater performance variation (5a1, 5c1, and 5e1) than after gastric bypass (5a2, 5c2, and 5e2), for which there seemed to be limited improvement potential given the variance smaller than or equal to 0.002. When the threshold for adequate weight loss was raised from 20 to 25% TWL, in line with previous recommendations [25, 26], the variance was at least 0.002 for all %TWL QIs. Consequently, the %TWL indicators were adjusted to achieving ≥ 25% TWL.

**Table 3:** Scores on the different quality indicators and the variability in outcomes

**Quality indicator**	**Median (%)**	**Range (min–max)**	**Variance**^**+**^** 2022**	**Variance**^**+**^** 2023**	Outliers*
**QI1** (Textbook Outcome)	94.5	86.6–98.5	0.0007	0.0009	Yes
**QI2** (Improved HbA1c)	99.2	88.2–100	0.002	0.002	
**QI3** (Follow-up)					
**3a** (1 st year)	96.9	45.5–100	0.01	0.006	
**3c** (3rd year)	69.5	37.2–88.3	0.01	0.01	
**3e** (5 th year)	54.1	41.5–65.9	0.02	0.02	
**QI4** (Complicated course)					
**4a** *(primary surgery)*	1.2	0.2–2.6	0.0004	0.0004	Yes
**4b** *(secondary surgery)*	1.8	0.0–12.1	0.0009	0.001	Yes
**QI5** (%TWL)					
**5a** (1 st year)					
**5a1** *(gastric sleeve)*	87.4	55.6–100	0.01	0.007	
**5a2** *(gastric bypass)*	97.2	87.8–100	0.0007	0.0001	No
**5c** (3rd year)					
**5c1** *(gastric sleeve)*	74.5	50.0–100	0.01	0.01	
**5c2** *(gastric bypass)*	91.7	81.7–98.4	0.002	0.001	
**5e** (5 th year)					
**5e1** *(gastric sleeve)*	63.9	40.0–100	0.02	0.02	
**5e2** *(gastric bypass)*	85.1	77.7–91.7	0.002	0.001	

*QI* = quality indicator, median = determined by merged data from 2022-2023, outlier = significantly deviating performance according to the funnel plot, ^+^ = two decimals unless variance is < 0.01, * = only displayed if variance is < 0.001, *HbA1c* = glycated hemoglobin, textbook outcome = composite endpoint for no complications within 30 days after surgery, hospital discharge within 2 days, and no readmission within 30 days; complicated course = postoperative complication Clavien-Dindo grade 3+ within the first 30 days, %TWL = percentage total weight loss, gastric bypass = Roux-en-Y gastric bypass, one anastomosis gastric bypass, or ring augmented gastric bypass

### Actionability

**Fig. 1:**
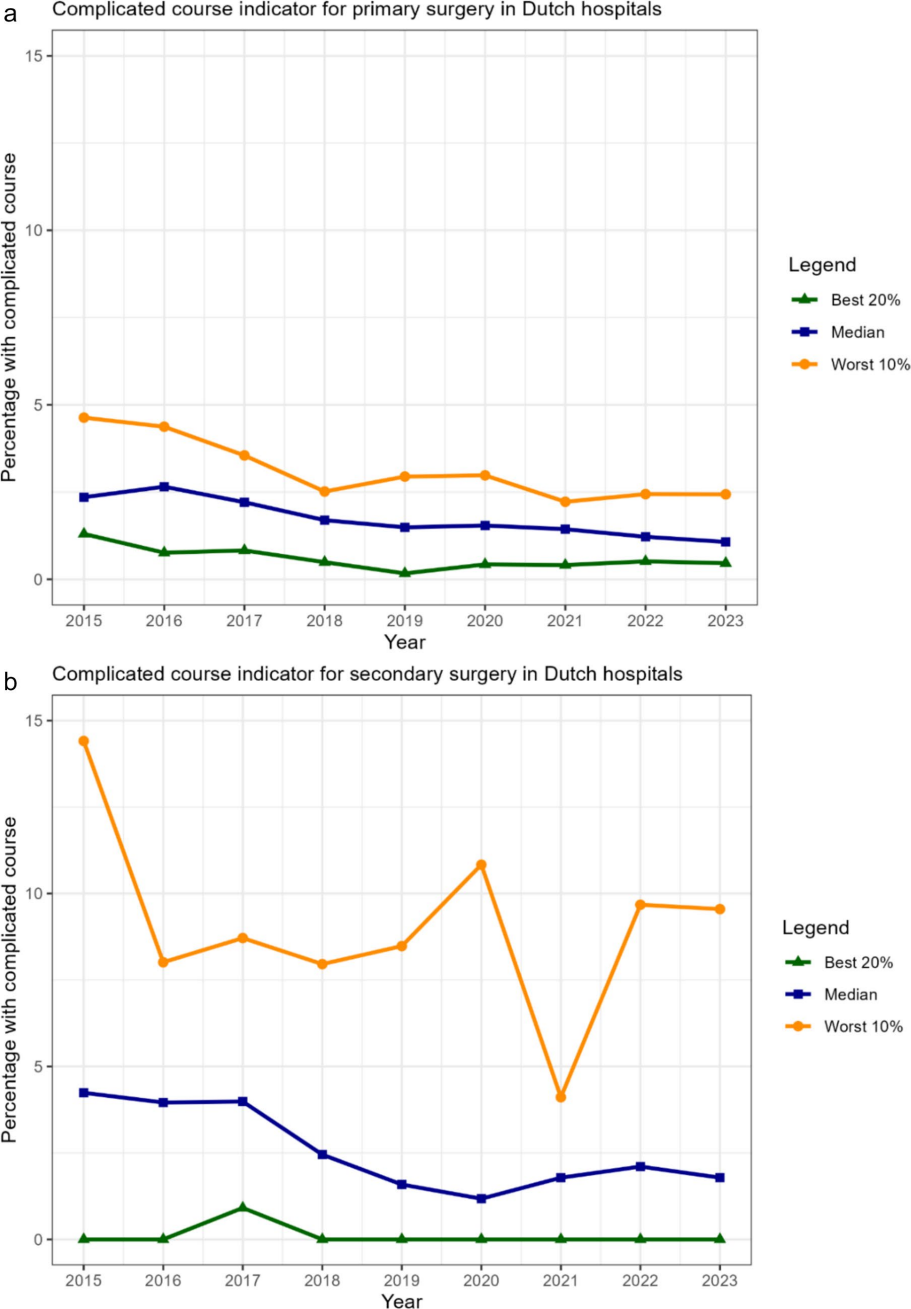
**a** Trends in hospital performance on the quality indicator for patients experiencing a complicated course after primary surgery. **b** Trends in hospital performance on the quality indicator for patients experiencing a complicated course after secondary surgery

The two QIs for complicated postoperative course after primary and secondary surgery demonstrated substantial improvement over time (see Figure 1a and 1b). For primary surgery, the median complication rate improved from 2.4% in 2015 to 1.1% in 2023, while for secondary surgery, the rate improved from 4.2 to 1.8%. In general, improvement was most pronounced in the groups with the worst performance, suggesting that these hospitals may have changed their care considerably based on their QI results (see supplementary figures 2a-2 g). There were no QIs where performance deteriorated, although the QIs for follow-up percentages at the 1^st^ and 3^rd^ year showed a significant decline for some hospitals during the years 2020 and 2021, coinciding with the COVID-19 pandemic (supplementary figures 2c and 2 d).

### Construct validity

For the confirmatory factor analysis, the 20% TWL QIs were adjusted to the proposed 25% TWL indicators, so that the reliability and consistency of the final QI set could be determined. Cronbach’s alphas were 0.53 (safety), 0.70 (treatment effectiveness), and 0.43 (completeness of follow-up), indicating that the three constructs were measured with sufficient consistency and reliability.

## Discussion

This systematic evaluation of the publicly available QI set for MBS in the Netherlands led to the proposal of an improved and more concise QI set without losing any insights regarding hospital performance on quality of MBS care. The follow-up and %TWL indicators at 2 and 4 years were so highly correlated with other years that they could be discontinued to reduce administrative burden, as they would not provide additional starting points for further improvement. Rather than eliminating the 20% TWL QIs due to insufficient improvement potential, they were adjusted to 25% TWL to enhance inter-hospital variance. Multiple QIs displayed moderate improvement over the years, most profound in the QIs examining complicated postoperative course. Overall, 13 of the initial 24 QIs remained in the final proposed QI set to measure safety, treatment effectiveness, and follow-up processes in MBS care.

With the increasing number of quality registries worldwide [9, 27], new or refined QI sets are frequently introduced. In MBS literature, methods have been described for composing new QI sets [10, 11], mostly covering an approach based on expert opinion. As outcomes and insights may change over time, periodic evaluation is crucial to maintain the added value of QI sets, and the importance of a systematic assessment should be emphasized. However, in MBS literature, this issue hardly receives attention, and to our knowledge, no study has reported on a systematic evaluation of QI sets for MBS. Periodic evaluation ensures the relevance of the QI set by assessing the added value of individual QIs, thereby preventing burdensome data registration that does not contribute to improving healthcare. Aside from attesting the merit of QIs which are already in use, performing a factor analysis helps in examining whether all relevant aspects in MBS care are adequately addressed and if all quality of care domains are sufficiently covered (e.g., patient-centeredness or timeliness of care). Thereby, the method used in this study does not only evaluate existing QIs but also stimulates discussions on areas where new QIs should be developed to add insights to the QI set.

QIs are most useful if they show significant varying performance between hospitals, so that hospitals and caregivers start searching for explanations when they are underperforming. As the 20% TWL QI showed very little variance, especially for the gastric bypass sub-indicators, the threshold for adequate weight loss was elevated to 25% TWL instead of abolishing the 20% TWL QI. That way, the current method is not only a tool to make the QI set more concise, but also to adjust QIs when they are still considered relevant based on the indicator content. This adjustment increased the inter-hospital variance, potentially renewing the incentive for hospitals to improve their weight loss results. Although the variance did not increase for the sleeve gastrectomy sub-indicators, these thresholds were elevated as well in the spirit of continuous quality improvement and striving for better results in general.

Quality registries aid in monitoring hospital performance and QI sets should meet this purpose without causing undue registration burden. Previous research showed that on average 52 min per working day are spent by healthcare professionals on entering information for quality registries [28]. When creating QI sets, getting the right balance between cost, i.e., withholding caregiver’s time from the patient, and benefit, i.e., potential quality improvement, should be considered [16]. A systematic assessment of QI sets may serve this cause, and the current study demonstrated that the systematic evaluation of the QI set is feasible and applicable to MBS care. These results imply that evaluating QI sets by applying this method should be considered in any MBS registry to ensure that QIs remain useful in clinical practice.

Some limitations should be noted. First, the statistical uncertainty associated with the hospital’s QI scores was not considered. The scores of low-volume hospitals will have higher statistical uncertainty because they are based on fewer observations, but with the present method, they get the same weight as results from high-volume hospitals. However, the question is whether this may have impacted the results. QIs such as improvement in HbA1c (determined in diabetic patients) and experiencing a complicated postoperative course after secondary surgery are calculated in subgroups of patients and thereby based on smaller sample sizes. As for parsimony and relevance the correlation coefficients for these QIs were much smaller than 0.7, it is unlikely that they would change to be above 0.7 if larger sample sizes had been considered. Regarding actionability, in general, the trends followed consistent patterns, and for usability, the variance was complemented by outlier identification in a funnel plot, in which hospital caseload is considered. Therefore, it seems unlikely that the results of the current study would change if statistical uncertainty had been incorporated. However, when applying the method in other quality registries with lower case volumes, caution is warranted, and it may then be desirable to include confidence intervals in the evaluation method. Second, even if a QI does not show any performance variability, its inclusion in the QI set may still be necessary, for instance, if mandated by governmental institutions. Third, had the evaluation been performed in another year, it may have yielded different results, e.g., because new QIs may have been introduced or when the performance of hospitals would have changed. Fourth, although a strength of the current method is the focus on the QI set, thereby surpassing traditional approaches that analyze QIs separately, the QIs could still have been evaluated on other criteria such as reliability and validity [12]. It may be interesting to incorporate these criteria in the current method as well. Future research may also focus on repeating the same analyses in other MBS registries and in registries with smaller caseloads to explore whether the method yields consistent added value. Additionally, it would be of interest to investigate whether other registries measure the same constructs.

## Conclusion

Following a systematic evaluation, the DATO QI set could be refined by modifying 6 indicators and removing another 11, leaving 13 QIs in the final proposed set without compromising the essential information needed to initiate quality improvement initiatives. Since QIs are typically introduced based on expert opinion, it is desirable to systematically evaluate the properties of the QI set. The reduction achieved in this study—eliminating nearly half of the initial QIs—shows that there are opportunities to decrease the registration burden for healthcare professionals in MBS care.

## Supplementary Information

Below is the link to the electronic supplementary material.

Supplementary file1 (DOCX 2295 KB)

## Data Availability

The data used for the analyses has not been made available but can be made accessible upon reasonable request to the corresponding author. Competing Interests The authors declare no competing interests.
