# Supplementary material for: Refining the Quality Indicator Set from the Dutch Audit for Treatment of Obesity to Ensure Usefulness in Clinical Practice
Source: Obes Surg. 2025 Jun 12;35(7):2616–25. doi: 10.1007/s11695-025-07898-2 (PMC12270963; doi:10.1007/s11695-025-07898-2)

Supplementary figure 1a. Funnel plot showing hospital performance on complicated course for primary surgery, 2023
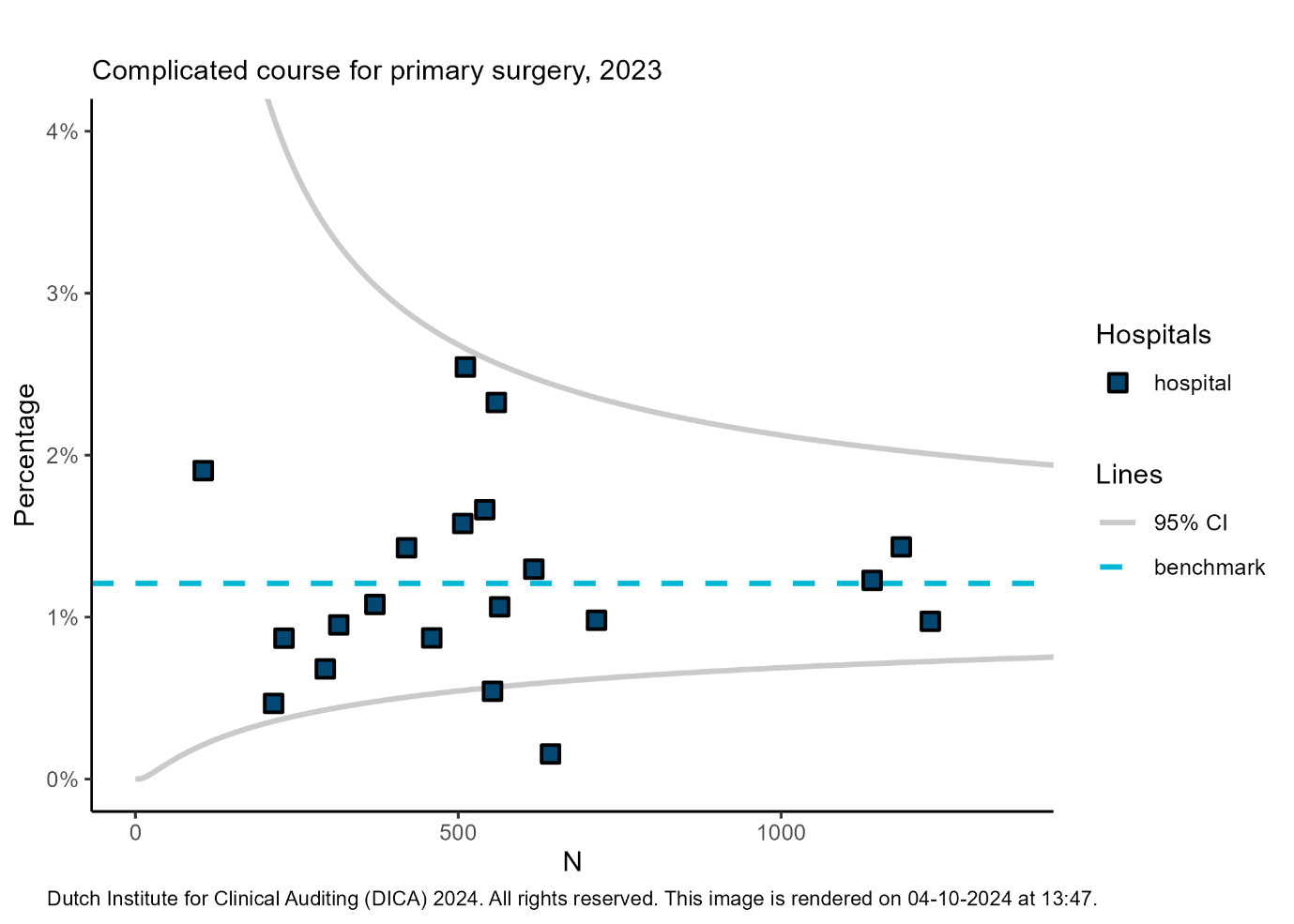


Complicated course = complication grade 3 according to the Clavien-Dindo classification within the first 30 days after surgery, N = number of patients operated per hospital, CI = confidence interval, benchmark = nationwide mean

Supplementary figure 1b. Funnel plot showing hospital performance on complicated course for secondary surgery, 2023
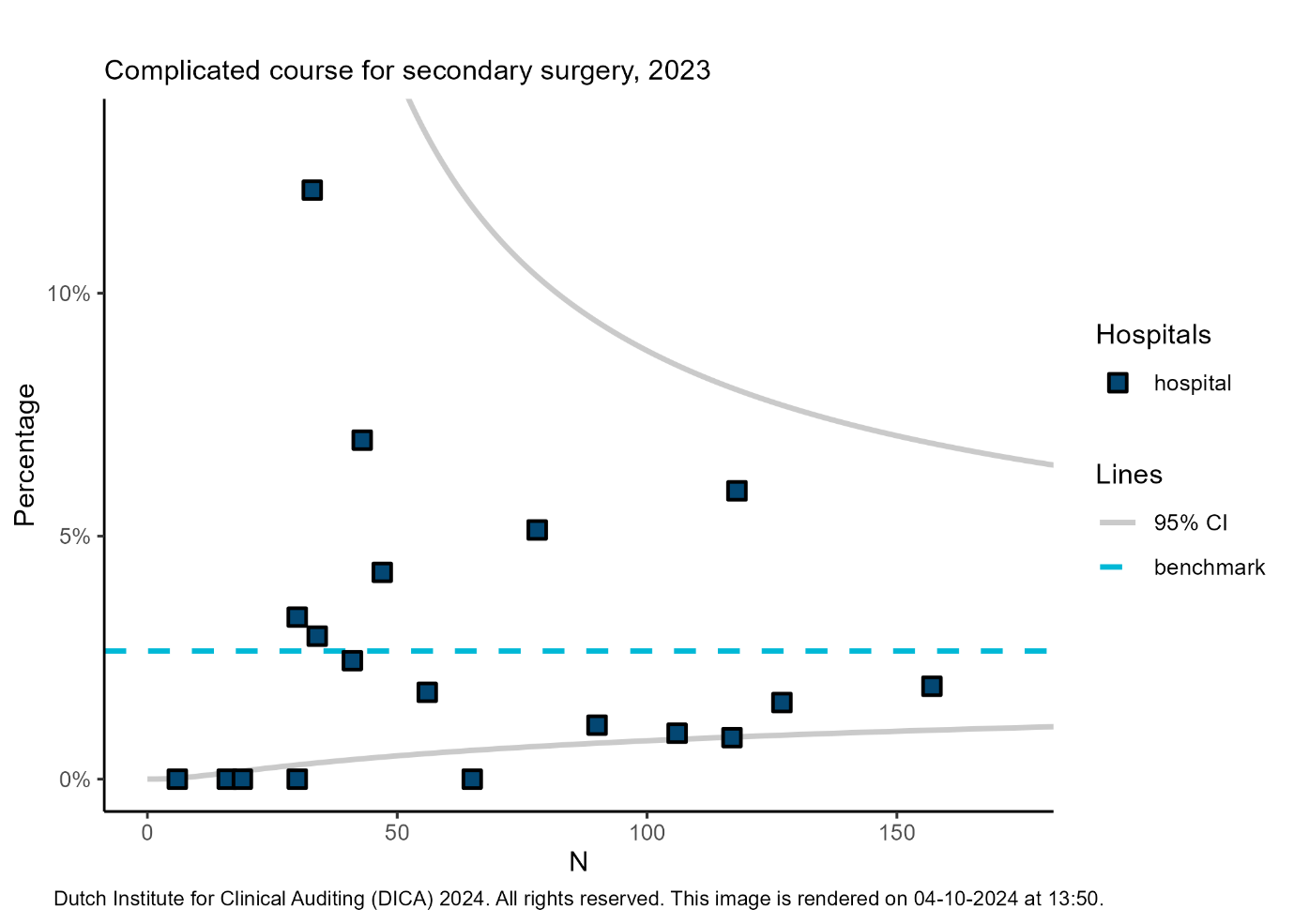


Complicated course = complication grade 3 according to the Clavien-Dindo classification within the first 30 days after surgery, N = number of patients operated per hospital, CI = confidence interval, benchmark = nationwide mean


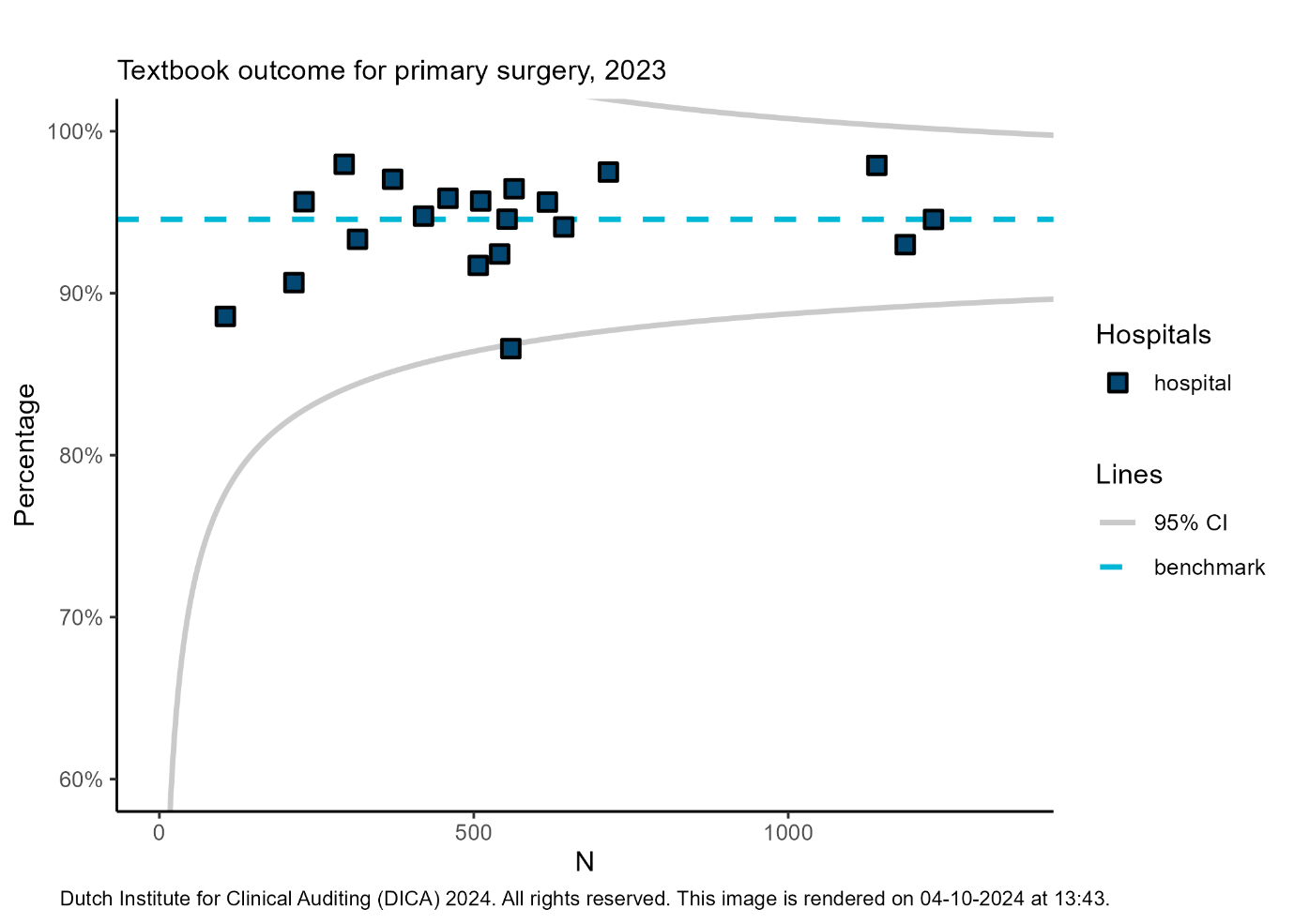
Supplementary figure 1c. Funnel plot showing hospital performance on textbook outcome for primary surgery, 2023

Textbook Outcome = composite endpoint for no complications within 30 days after surgery, hospital discharge within 2 days, and no readmission within 30 days, N = number of patients operated per hospital, CI = confidence interval, benchmark = nationwide mean

Supplementary figure 1d. Funnel plot showing hospital performance on achieving at least 20% total weight loss, 1 year after primary gastric bypass, 2023


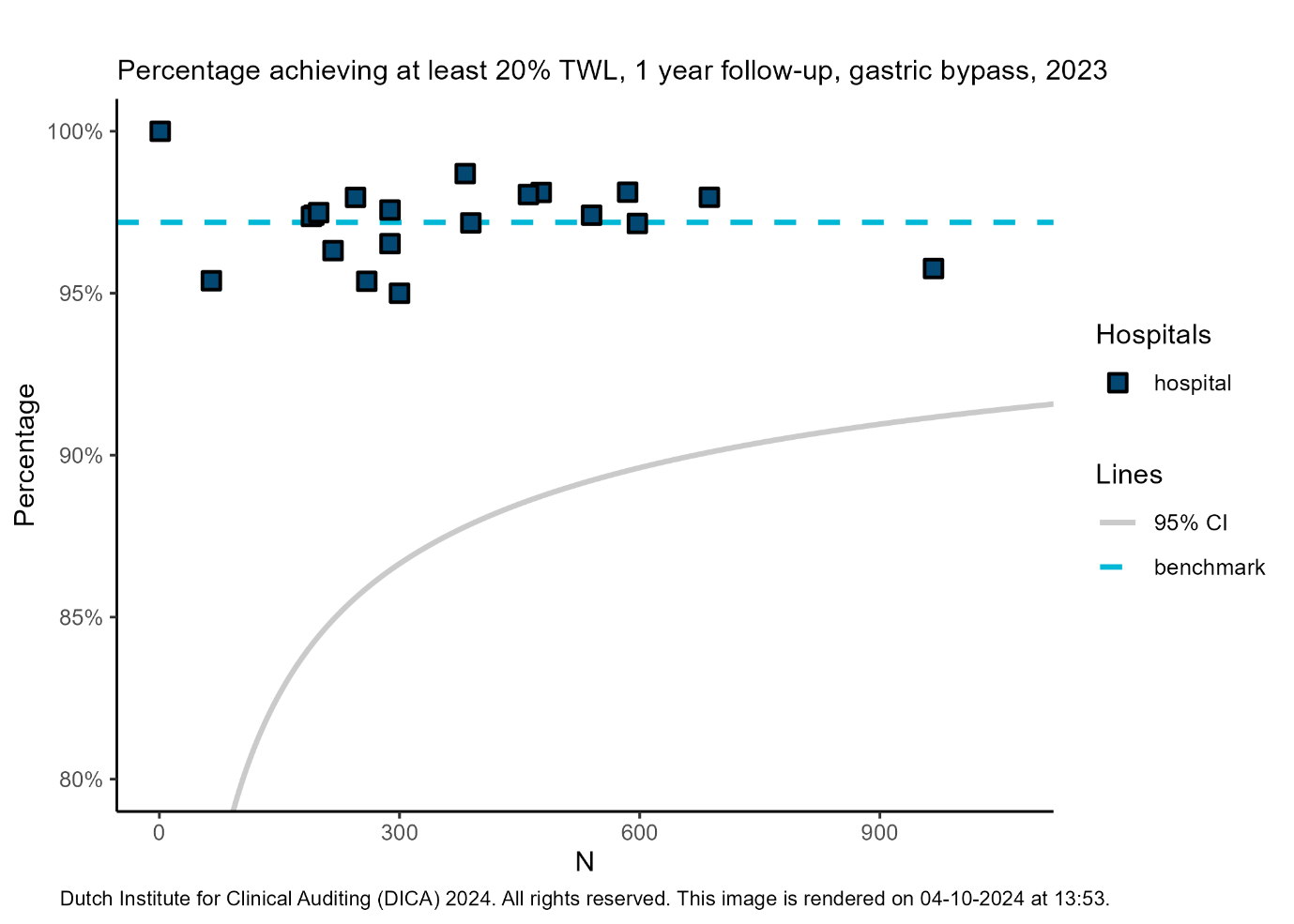


TWL = total weight loss, N = number of patients with available weight, CI = confidence interval, benchmark = nationwide mean

Supplementary figure 2a-g. Performance trends for Dutch hospitals on certain quality indicators since their introduction. 2a


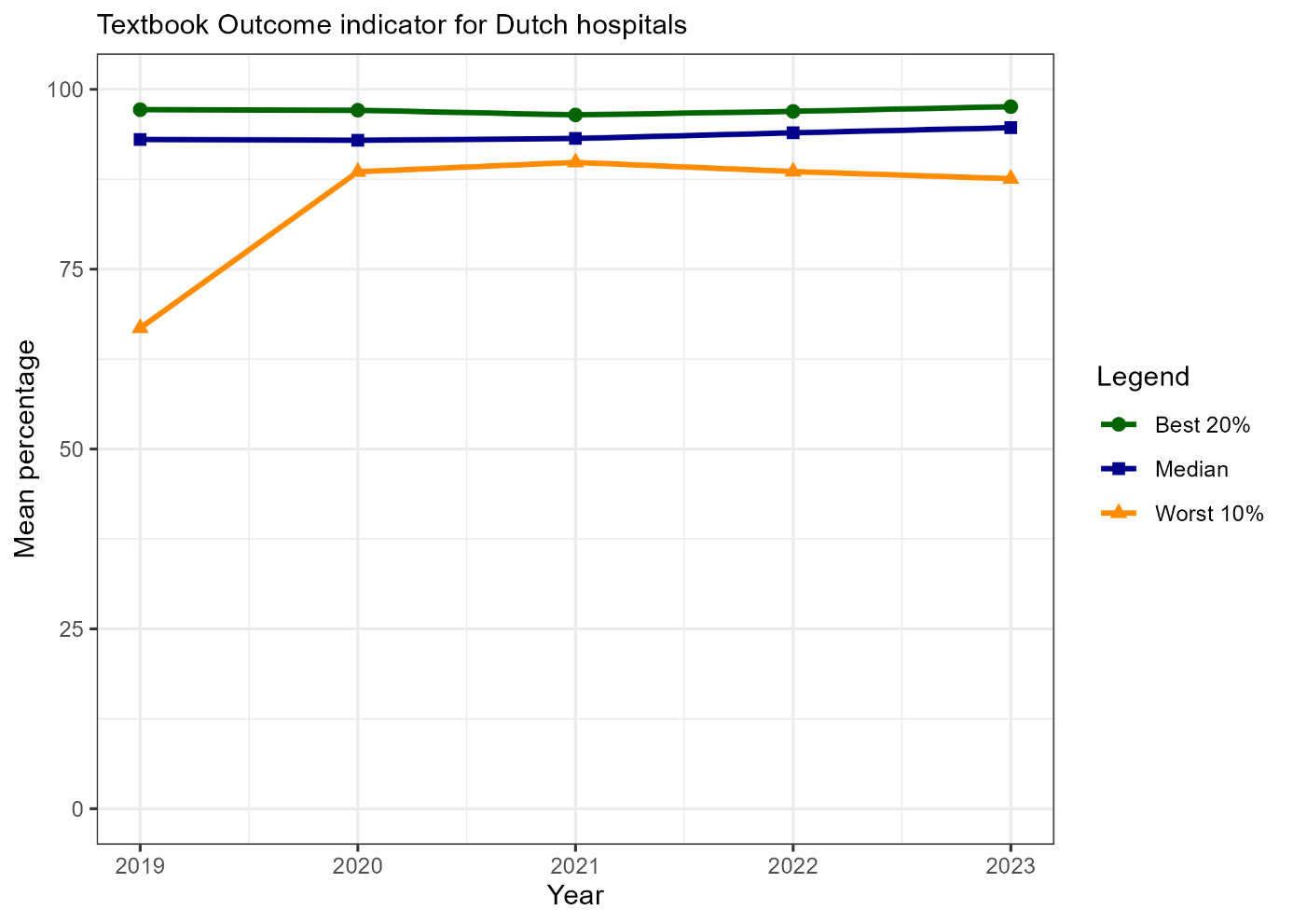


Supplementary figure 2b.
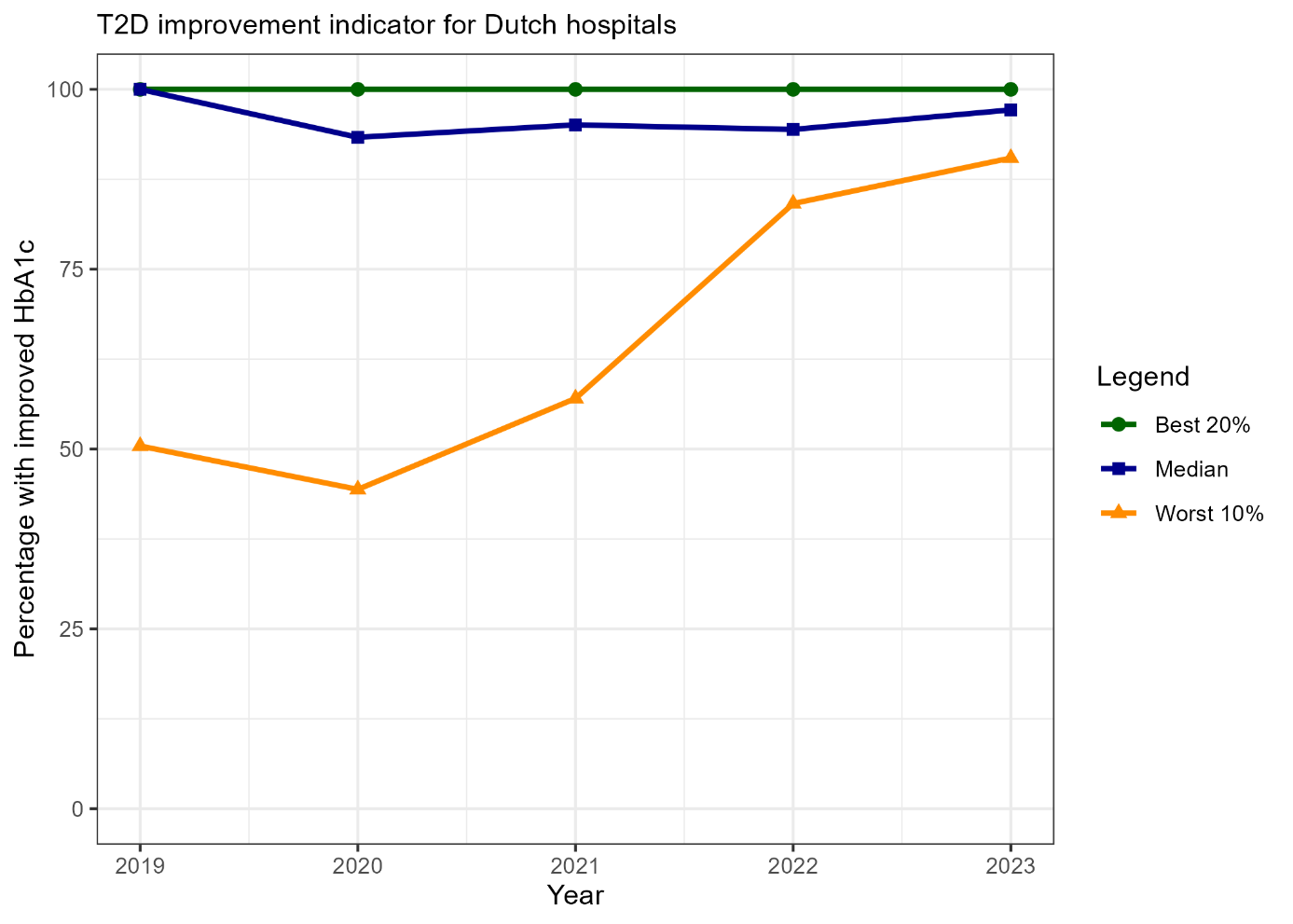


Supplementary figure 2c.
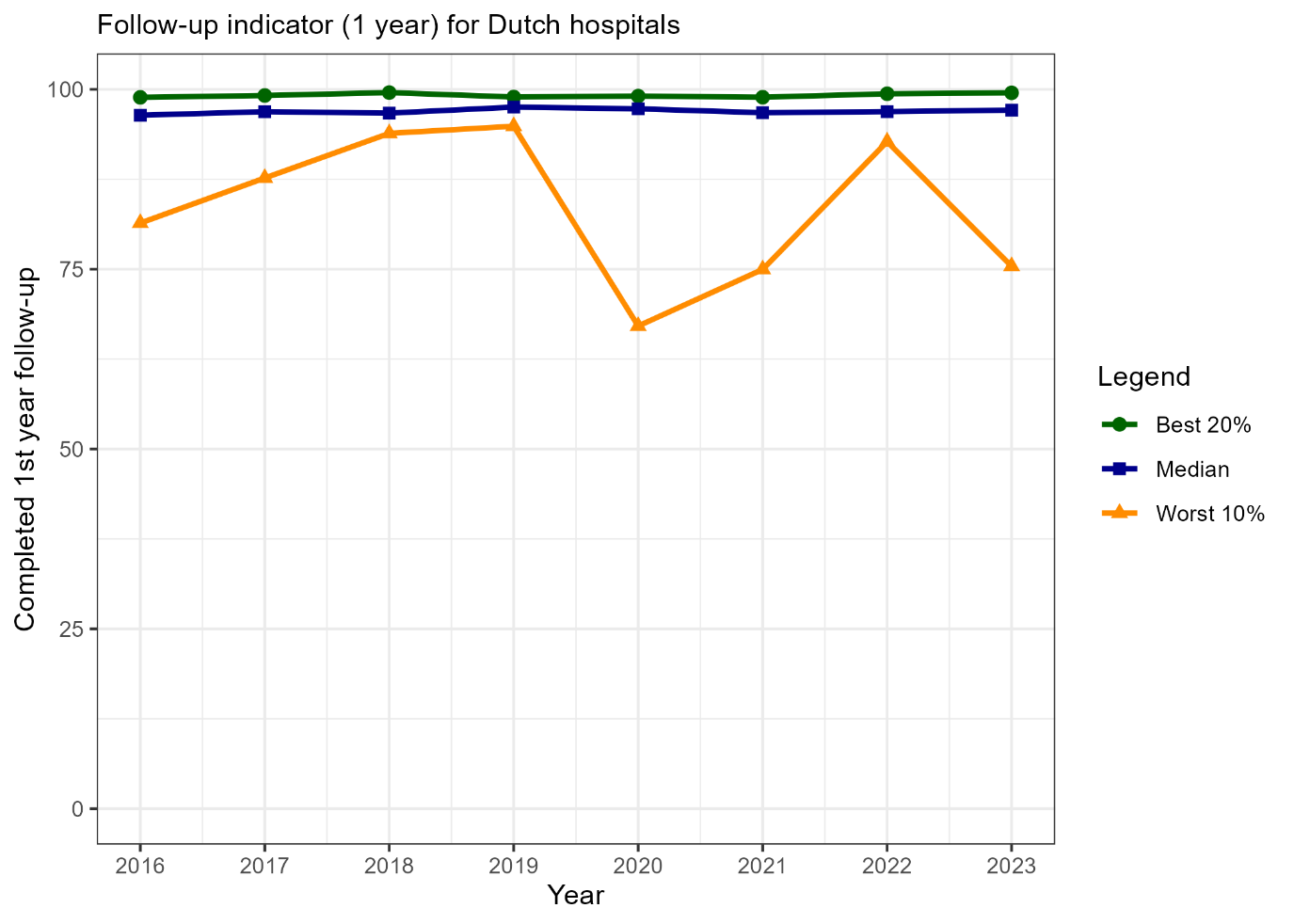


Supplementary figure 2d.


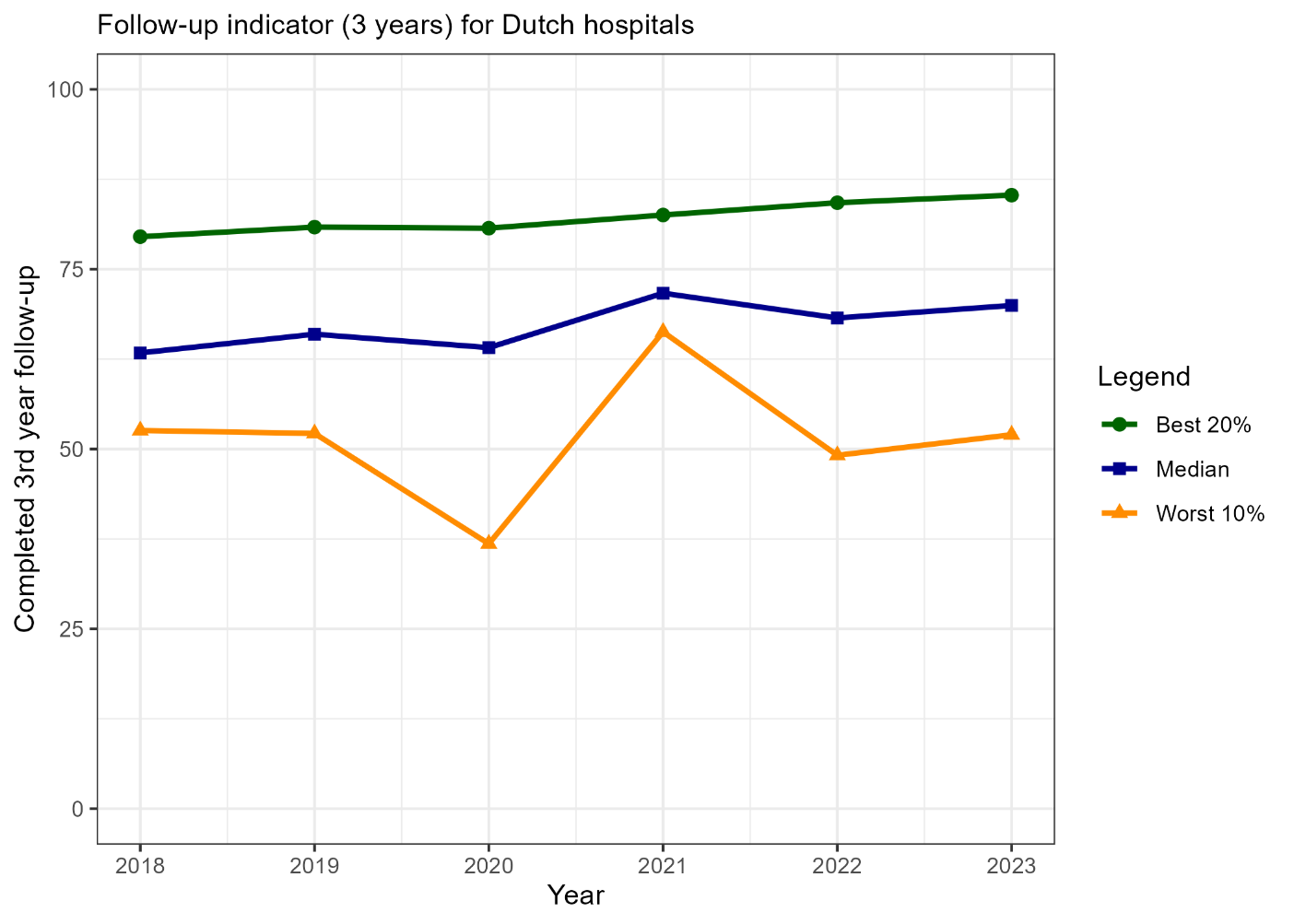


Supplementary figure 2e.


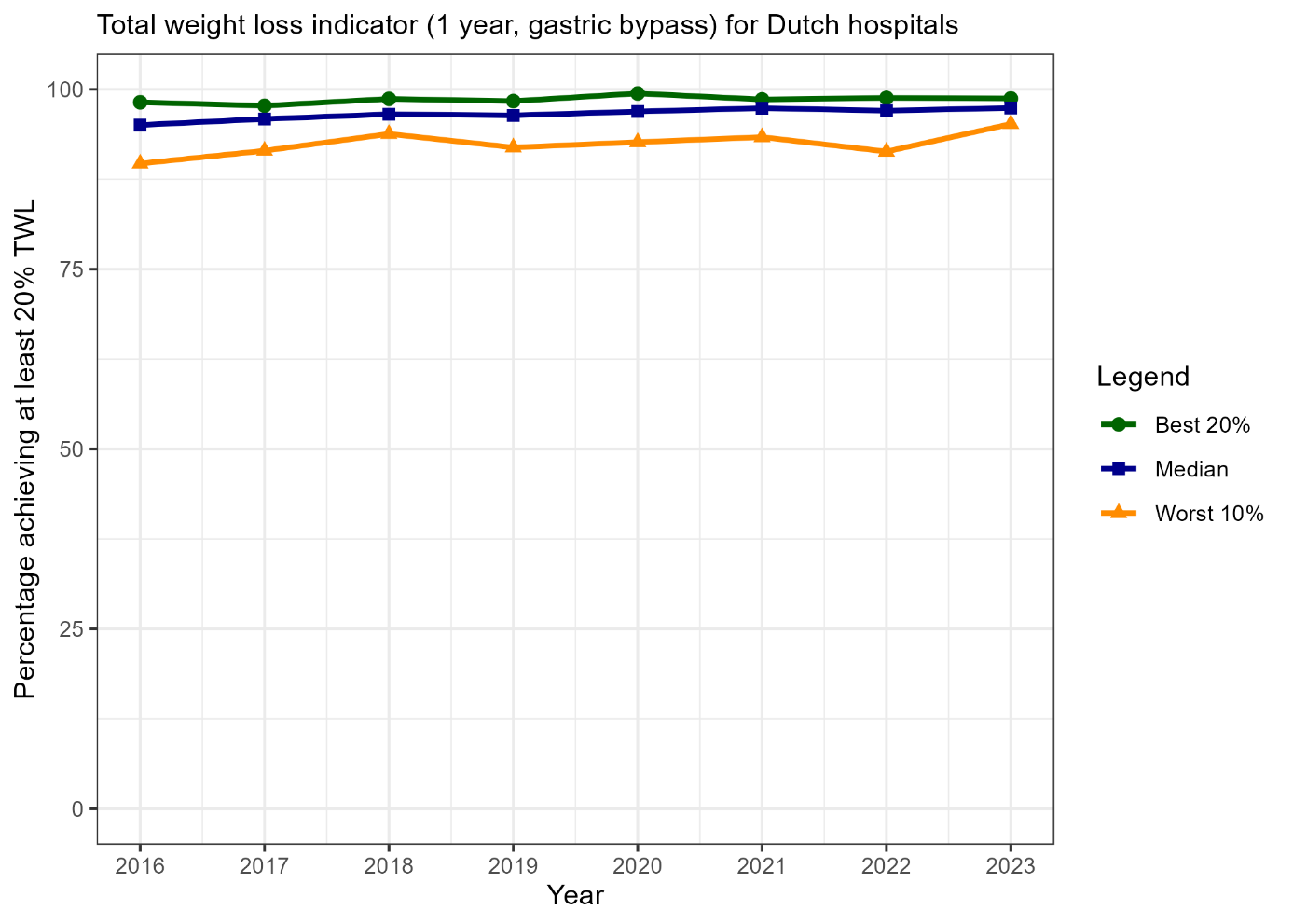


Supplementary figure 2f.


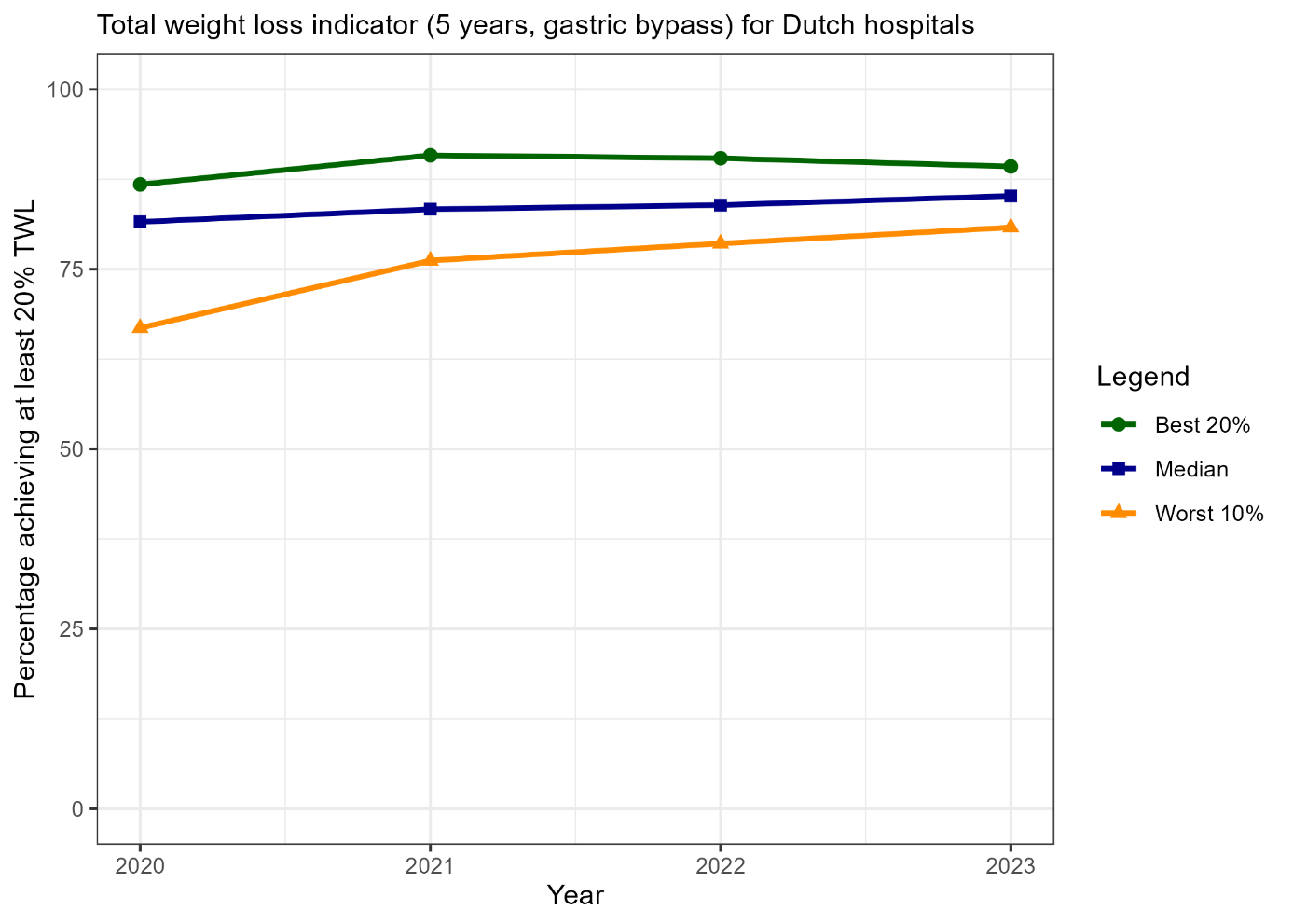


Supplementary figure 2g.


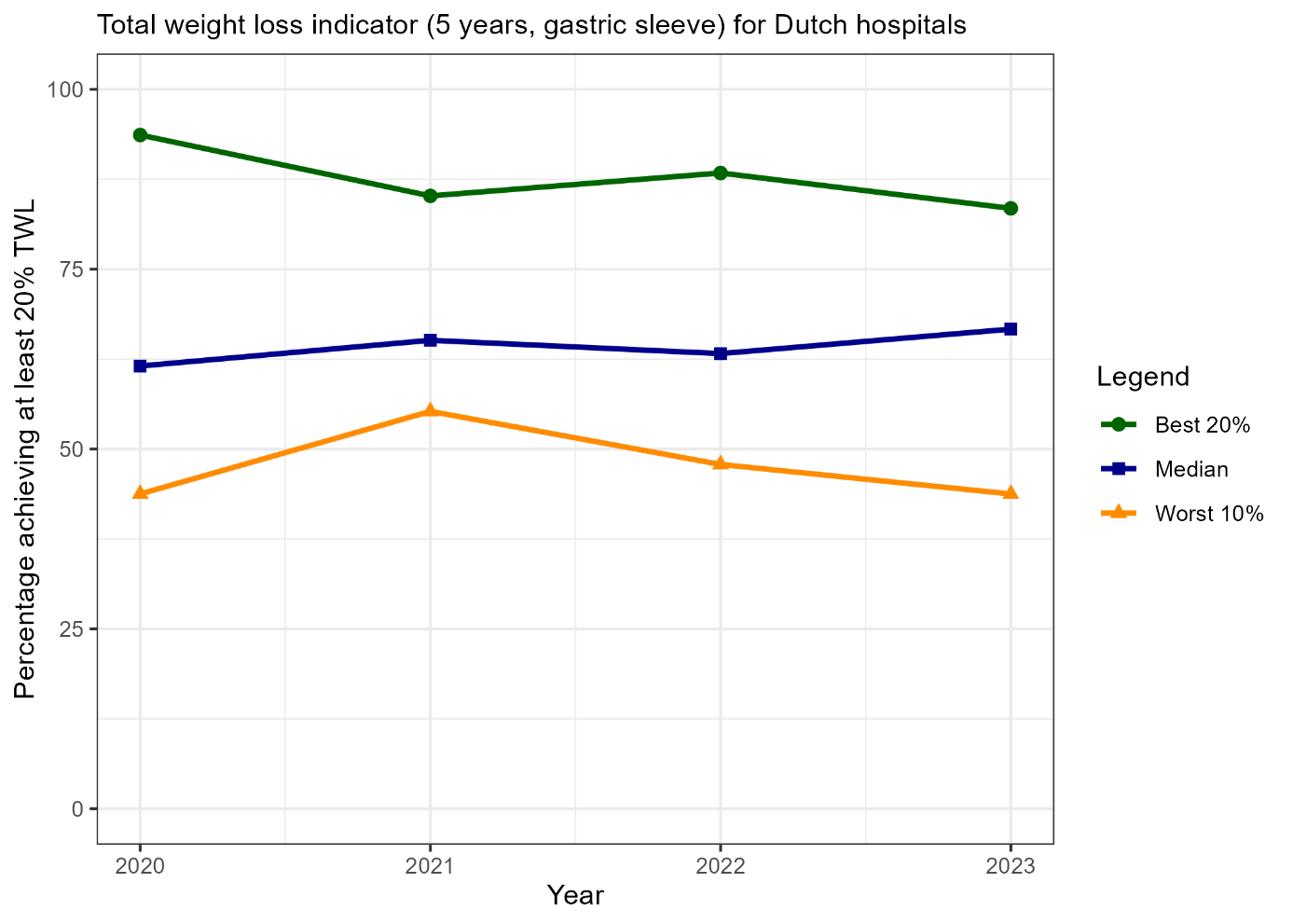

Supplement: Supplementary file 1 — Supplementary file1 (DOCX 2295 KB) [file 11695_2025_7898_MOESM1_ESM.docx]
